# Time trend and costs of hospitalizations with diabetes mellitus as main diagnosis in the Brazilian National Health System, 2011 to 2019

**DOI:** 10.1590/S2237-96222023000400006.en

**Published:** 2024-01-05

**Authors:** Ludmilla Ferreira da Costa, Taisa Lara Sampaio, Lenildo de Moura, Roger dos Santos Rosa, Betine Pinto Moehlecke Iser

**Affiliations:** 1 Universidade do Sul de Santa Catarina, Curso de graduação em Medicina, Tubarão, SC, Brazil; 2 Pan-American Health Organization, Coordenação de Doenças Crônicas Não Transmissíveis e Saúde Mental, Asunción, Departamento Central, Paraguay; 3 Universidade Federal do Rio Grande do Sul, Departamento de Medicina Social, Porto Alegre, RS, Brazil; 4 Universidade do Sul de Santa Catarina, Programa de Pós-Graduação em Ciências da Saúde, Tubarão, SC, Brazil

**Keywords:** Diabetes Mellitus, Hospitalization, Hospital Costs, Brazilian National Health System, Population Studies in Public Health, Time Series Studies, Diabetes Mellitus, Hospitalización, gastos Hospitalarios, Sistema Único de Salud, Estudios Poblacionales en Salud Pública, Estudios de Series Temporales, Diabetes Mellitus, Hospitalização, Custos Hospitalares, Sistema Único de Saúde, Estudos Populacionais em Saúde Pública, Estudos de Séries Temporais

## Abstract

**Objective:**

To analyze the diabetes mellitus (DM) temporal trend and hospitalization costs in Brazil, by region, Federative Units (FUs) and population characteristics, from 2011 to 2019.

**Methods:**

This was an ecological study with data from the Hospital Information System, analyzing the annual trend in hospitalization rates for DM according to sex, age, race/skin color and region/FU by Prais-Winsten generalized linear regression.

**Results:**

A total of 1,239,574 DM hospitalizations were recorded in the country and the hospitalization rates was 6.77/10,000 inhabitants in the period. The DM hospitalization rates trend was falling for both sexes and in most regions, while it was rising in the younger population and for length of stay (average 6.17 days). Total expenditure was US$ 420,692.23 and it showed a rising trend.

**Conclusion:**

The temporal trend of hospitalization rates due to DM was falling, with differences according to region/FU and age group. Average length of stay and expenditure showed a rising trend.

## Study contributions

**Main results:**

From 2011 to 2019, the diabetes *mellitus* hospitalization rate was 6.77 per 10,000 inhabitants, with a falling trend. Total expenditure was US$420,692.23 and it showed a rising trend.

**Implications for services:**

The study warns of the increase in child and adolescent hospitalizations, which indicates the need to invest in preventive actions and early diagnosis.

**Perspectives:**

The increase in length of hospital stay and related costs indicates a worrying scenario for the Brazilian National Health System and emphasizes the need to improve access to and quality of care, with a focus on diabetes education, so as to avoid complications and hospitalizations.

## RESUMEN

**Objetivo:**

Analizar la tendencia temporal y los costos de las internaciones por diabetes mellitus (DM) en Brasil, por región, Unidades Federativas (UF) y características poblacionales, de 2011 a 2019.

**Métodos:**

Estudio ecológico con datos del Sistema de Información Hospitalaria, analizando la tendencia anual de las tasas de hospitalización (TH) por DM según sexo, edad, raza/color y región/UF mediante regresión de Prais-Winsten.

**Resultados:**

Se registraron 1.239.574 hospitalizaciones por DM en el país y la TH fue de 6,77/10 mil habitantes en el período. La tendencia de TH por DM fue decreciente en ambos sexos y en la mayoría de las regiones y creciente en la población más joven y en la duración de la estancia. El gasto total fue de 420.692.238 dólares y fue creciente.

**Conclusión:**

La tendencia temporal de TH por DM fue decreciente, con diferencias según región/UF y grupo de edad. La duración media de la estancia y los gastos mostraron una tendencia creciente.

## RESUMO

**Objetivo:**

Analisar a tendência temporal e gastos das internações por diabetes *mellitus* (DM) no Brasil, por região, Unidades da Federação (UFs) e características populacionais, de 2011 a 2019.

**Métodos:**

Estudo ecológico com dados do Sistema de Informações Hospitalares, analisando a tendência anual das taxas de internação (TIs) por DM segundo sexo, idade, raça/cor e região/UF pela regressão linear generalizada de Prais-Winsten.

**Resultados:**

Foram registradas 1.239.574 internações por DM no país e a TI foi de 6,77/10 mil habitantes, no período. A tendência das TIs por DM foi decrescente nos dois sexos e na maioria das regiões, e crescente na população mais jovem e no tempo de internação (média de 6,17 dias). O gasto total foi de US$ 420.692,23 e apresentou tendência crescente.

**Conclusão:**

A tendência temporal da TI por DM foi decrescente, com diferenças segundo região/UF e grupo etário. O tempo médio de internação e os gastos apresentaram tendência de aumento.

## INTRODUCTION

The global outlook for diabetes *mellitus* (DM), published by the International Diabetes Federation (IDF) in 2019, revealed that 1.1 million children and adolescents were living with type 1 diabetes *mellitus* (DM) and around 463 million (9.3%) of adults aged between 20 and 79 years were living with DM, around 90% of whom had type 2 diabetes; it is also estimated that a further 232 million adults are undiagnosed.^1^ The global prevalence trend continues to rise, with a forecast of reaching 783 million people living with DM by 2045.^2^ Regarding the cost, worldwide US$ 760 billion have been allocated to diabetes annually, with a forecast of reaching US$ 1.05 trillion by 2045.^1,2^

In 2019, Brazil ranked 5^th^ worldwide in terms of adults living with diabetes, with 16.8 million or 7.9% of the population suffering from the disease;^1^ it is estimated there will be 26 million (11.3%) by 2045.^2^ Prevalence of type 1 DM (common up to 14 years of age) in 2019 was 51,500 while incidence was 7,300 cases/year, with a rising trend.^1^ In 2014, diabetes-related costs in Brazil amounted to US$ 264 million, with the average cost of each hospitalization being US$ 845.^3^ According to 2019 estimates by IDF, the total expenditure for diabetes in the country amounted to US$ 52.3 billion.^1^

This scenario of increasing incidence and prevalence of the disease throughout the world increases the need for hospital admissions, resulting from decompensated conditions and/or acute and chronic complications, which places this issue on the list of impacts on public health, justifying the scientific effort to clarify related topics.^4^ The objective of this study was to analyze the temporal trend and hospitalization costs for cases with diabetes *mellitus* as their main diagnosis in Brazil from 2011 to 2019, by region, FU and population characteristics.

## METHODS

We conducted a mixed exploratory ecological study, including time series analysis of DM hospitalization rates and related expenditure, and analysis of population subgroups, for Brazil and its regions/FUs, from 2011 to 2019. 

The study period was defined considering years prior to the COVID-19 pandemic, as data for 2020 and 2021 showed fluctuations, without a defined pattern. In addition, data for 2021 were still preliminary. 

The Brazilian population was estimated to be 210,147,125 inhabitants in 2019.^5^ Between 2011 and 2019, an overall total of 103,051,010 hospitalizations were recorded in Brazil.^6^ Brazilian National Health System (*Sistema Único de Saúde* - SUS) hospitalizations accounted for 69% of total hospitalizations in Brazil in 2019.^6^ This study included all hospitalizations registered on the SUS Hospital Information System (*Sistema de Informações Hospitalares do SUS* - SIH-SUS), between 2011 and 2019, for people resident in Brazil and whose main diagnosis was DM [codes E10 to E14 of the International Statistical Classification of Diseases and Related Health Problems (ICD10)]. The information was extracted from the SIH/SUS, on 3/14/2022 and 9/9/2022. Data on the Brazilian reference population were collected from the database of the Brazilian Institute of Geography and Statistics (*Instituto Brasileiro de Geografia e Estatística* - IBGE), accessed on 4/5/2022.

The variables defined for the study were: number of hospitalizations per year of care (main independent variable of the study), stratified according to Region and FU, age range, sex and race/skin color. 

Estimated cost of hospitalizations (in BRL) and average length of stay (days) (dependent variables) were extracted according to the year of care, obtaining the average data for the period for the regions/FUs. Monetary correction of the total amounts ​​in BRL was performed by applying the General Market Price Index (*Índice Geral de Preços ‒ Mercado*), using the Brazilian Central Bank calculator. In order to determine the value in United States dollars (US$), we used the average exchange rate for each year from 2011 to 2019, based on data held on the database of the Brazilian Institute for Applied Economic Research (*Instituto de Pesquisa Econômica Aplicada* - IPEA), access on 9/9/2022, thus obtaining an international parameter.

The hospitalization rate was calculated by dividing the total number of DM hospitalizations by the population in the same place and period, multiplied by 10,000 inhabitants. 

The diabetes *mellitus* hospitalization rates were stratified by:

- Sex (male and female);
- Age group: children and adolescents (up to 19 years), young adult (20-39 years), adult (40-59 years), young-old (60-79 years), oldest-old (80 years or over);
- Race/skin color: white, black, mixed race, asian and indigenous;
- Region/FU: North (Rondônia, Acre, Amazonas, Roraima, Pará, Amapá and Tocantins), Northeast (Maranhão, Piauí, Ceará, Rio Grande do Norte, Paraíba, Pernambuco, Alagoas, Sergipe and Bahia), Midwest (Mato Grosso do Sul, Mato Grosso, Goiás and Distrito Federal), South (Paraná, Santa Catarina and Rio Grande do Sul) and Southeast (São Paulo, Rio de Janeiro, Minas Gerais and Espírito Santo).

The hospitalization rate trend analysis was performed based on generalized linear regression using the Prais-Winsten method, which provided the coefficient of determination (R²). Annual percentage change (APC) was calculated taking the regression β coefficient according to the following formula: APC = (-1+10β)*100. We calculated 95% confidence intervals (95%CI) for APC. When performing statistical comparison of the proportions between the categories, we considered the average rates for the period and the 95%CIs. We adopted a 5% significance level. Data organization, processing and analysis, as well as organization of graphs and maps, was performed using an electronic spreadsheet. The statistical analysis was performed using Stata®12.0. 

The study was approved by the Research Ethics Committee of the *Universidade do Sul de Santa Catarina* (UNISUL), as per Opinion No. 5.166.919, and the research protocol is in accordance with the National Health Council resolutions.

## RESULTS

Between 2011 and 2019, 1,239,574 hospitalizations were recorded of cases whose main diagnosis was DM, representing 1.2% of total SUS hospitalizations (n ​​= 103,051,010). Over the period, the hospitalization rate due to DM in Brazil was 6.77/10,000 inhabitants, varying between 7.5/10,000 inhabitants in 2011 and 6.5/10,000 inhabitants in 2019, and it showed a falling trend (Table 1). 

**Table 1:** Hospitalization Rate (HR) average and trend for cases having Diabetes *Mellitus* as main diagnosis (per 10,000 inhabitants) in the Brazilian National Health System, by Brazilian Region and Federative Unit, 2011-2019

Region and Federative Unit	HR 2011	HR 2019	Average HR 2011-2019 (95%CI)	APC (95%CI)	P-value	Trend
**Brazil**	**7.55**	**6.48**	**6.77 (6.44;7.** **10)**	**-0.26 (-0.40;-0.09)**	**0.010**	**Falling**
**Northern Region**	**7.40**	**7.58**	**7.34 (7.12;7.57)**	**0.05 (-0.** **24;0.41)**	**0.800**	**Stationary**
Rondônia	13.24	10.06	11.80 (10.8;12.78)	-0.57 (-0.74;-0.28)	0.010	Falling
Acre	6.53	5.14	5.58 (5.13;6.03)	-0.31 (-0.51;-0.02)	0.040	Falling
Amazonas	4.85	6.99	5.86 (5.11;6.60)	0.91 (0.26;1.82)	0.010	Rising
Roraima	9.07	12.38	10.43 (9.19;11.68)	1.29 (-0.02;4.50)	0.060	Stationary
Pará	6.84	7.81	7.21 (6.91;7.51)	0.02 (-0.01;0.55)	0.060	Stationary
Amapá	6.33	3.29	4.58 (3.52;5.64)	-0.59 (-0.77;-0.28)	0.010	Falling
Tocantins	10.72	6.89	8.23 (6.84;9.63)	-0.70 (-0.87;-0.34)	0.100	Stationary
**Northeast Region**	**8.89**	**7.64**	**7.97 (7** **.57;8.36)**	**-0.29 (-0.42;-0.13)**	**0.010**	**Falling**
Maranhão	9.25	14.83	11.88 (10.31;13.47)	3.79 (2.39;5.76)	< 0.001	Rising
Piauí	12.20	10.27	11.89 (11.05;13.73)	-0.44 (-0.76;0.32)	0.170	Stationary
Ceará	6.56	5.58	5.34 (4.91;5.78)	6.76 (-0.96;-0.63)	< 0.001	Rising
Rio Grande do Norte	8.88	6.68	7.63 (7.00;8.27)	-0.38 (-0.77;0.66)	0.290	Stationary
Paraíba	9.05	6.74	7.58 (7.07;8.09)	-0.34 (-0.61;0.12)	0.110	Stationary
Pernambuco	8.51	5.76	6.43 (5.63;7.24)	-0.92 (-0.96;-0.82)	< 0.001	Falling
Alagoas	9.42	5.33	6.66 (5.55;7.76)	-0.68 (-0.84;-0.38)	0.060	Stationary
Sergipe	4.05	4.27	4.28 (3.72;4.83)	0.17 (-0.37;1.19)	0.540	Stationary
Bahia	10.19	7.61	8.93 (7.92;9.94)	-0.58 (-0.70;-0.40)	< 0.001	Falling
**Southeast Region**	**6.08**	**5.51**	**5.61 (5.40;5.82)**	**-0.48 (-0.67;-0.19)**	**0.010**	**Falling**
Minas Gerais	7.88	7.82	7.71 (7.48;7.94)	-0.07 (-0.22;-0.11)	0.390	Stationary
Espírito Santo	6.69	6.01	6.34 (6.08;6.61)	-0.21 (-0.29;-0.09)	< 0.001	Falling
Rio de Janeiro	5.59	4.52	4.69 (4.33;5.05)	-0.70 (-0.84;0.46)	< 0.001	Falling
São Paulo	5.37	4.78	4.91 (4.72;5.10)	-0.46 (-0.64;-0.19)	0.010	Falling
**Southern Region**	**8.73**	**6.81**	**7.60 (7.06;8.14)**	**-0.61 (-0.79;-** **0.31)**	**0.010**	**Falling**
Paraná	8.71	6.92	7.50 (6.82;8.18)	-0.41 (-0.68;0.10)	0.080	Stationary
Santa Catarina	7.01	6.22	6.52 (6.27;6.77)	0.17 (-0.28;0.02)	0.070	Stationary
Rio Grande do Sul	9.78	7.08	8.40 (7.65;9.15)	-0.55 (-0.59;-0.50)	< 0.001	Falling
**Midwest Region**	**8.73**	**5.78**	**6.77 (5.91;6.63)**	**-0.87** **(-0.92;-0.81)**	**< 0.001**	**Falling**
Mato Grosso do Sul	7.36	7.40	7.02 (6.66;7.38)	0.00 (-0.52;1.09)	0.100	Stationary
Mato Grosso	8.90	5.77	7.16 (6.26;8.05)	-0.61 (-0.68;-0.53)	< 0.001	Falling
Goiás	9.76	5.22	6.84 (5.54;8.15)	-0.74 (-0.86;-0.53)	< 0.001	Falling
**Distrito Federal**	**7.46**	**5.59**	**5** **.88 (5.17;6.59)**	**8.33 (-0.96;-0.69)**	**<** **0.001**	**Rising**

HR: Hospitalization rate (per 10,000 inhabitants); 95%CI: 95% confidence interval; APC: Annual percentage change.

**Figure 1:**
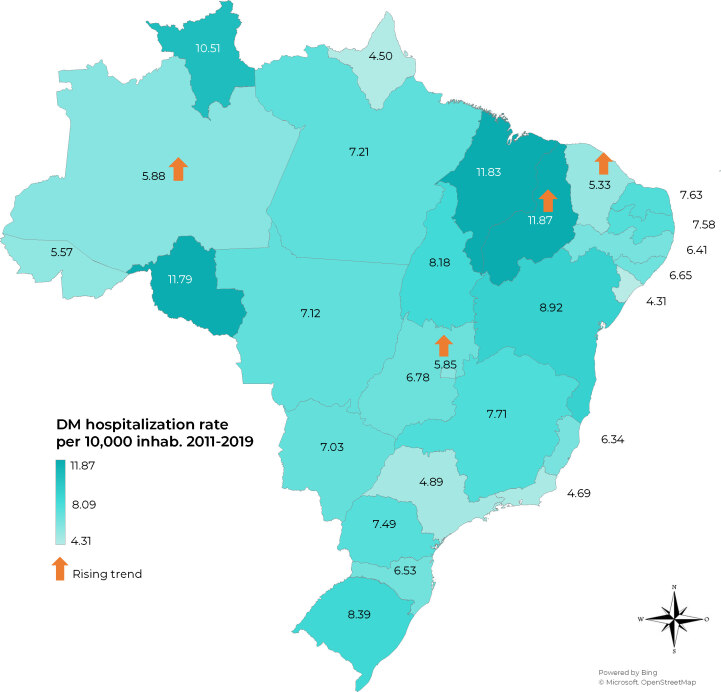
Choropleth map of the average hospitalization rate for cases having diabetes *mellitus* as main diagnosis in the Brazilian National Health System by Federative Unit (per 10,000 inhabitants) and indicative of rising trend for the period 2011-2019

Among the regions, the Northeast had the highest hospitalization rate due to DM (7.97/10,000), while the Southeast region had the lowest hospitalization rate (5.61/10,000). The trend was stationary in the Northern region and falling in the other regions. Analysis of the hospitalization rate by FU showed that the states of Piauí, Maranhão, Rondônia and Roraima presented a hospitalization rate above 10 per 1,000, this being above the national hospitalization rate due to DM (Figure 1). There was a rising hospitalization rate trend in the states of Amazonas, Maranhão, Ceará and Distrito Federal and a falling hospitalization rate trend in 11 FUs. Table 1 shows the hospitalization rate at the beginning and end of the study period by region and FU.

In relation to sex, the hospitalization rate due to DM in Brazil as a whole was 7.12/10,000 inhabitants for females and 6.43/10,000 inhabitants for males. In the Northeast and Southern regions the hospitalization rates were above the national average, for females and males, respectively. There was a falling hospitalization rate trend for both sexes. 

Analysis by age group showed an increase in the hospitalization rate as age increased. The hospitalization rate in the child and adolescent age group was 1.35 per thousand and it exceeded the rate of 30.31 hospitalizations/10,000 from 60 years of age upwards. There was a rising hospitalization rate temporal trend among children and adolescents aged 5 to 14 years and a stationary trend among those aged between 15 and 19. In the young adult group (20 to 24 years old) and in the adult group (45 to 59 years old), the trend was stationary. There was a falling trend in the other age groups. 

With regard to race/skin color, those of mixed race (average of 211 hospitalizations/10,000) had the highest hospitalization rates and were the only group in this category that showed a rising trend in the period. It must be emphasized that records with no information on race/skin color accounted for 30.4% of the total, however over the period there was a reduction in the percentage of records for which this information was missing, which was accompanied by an increase in the number of hospitalizations in the mixed race/skin color group.

**Figure 2:**
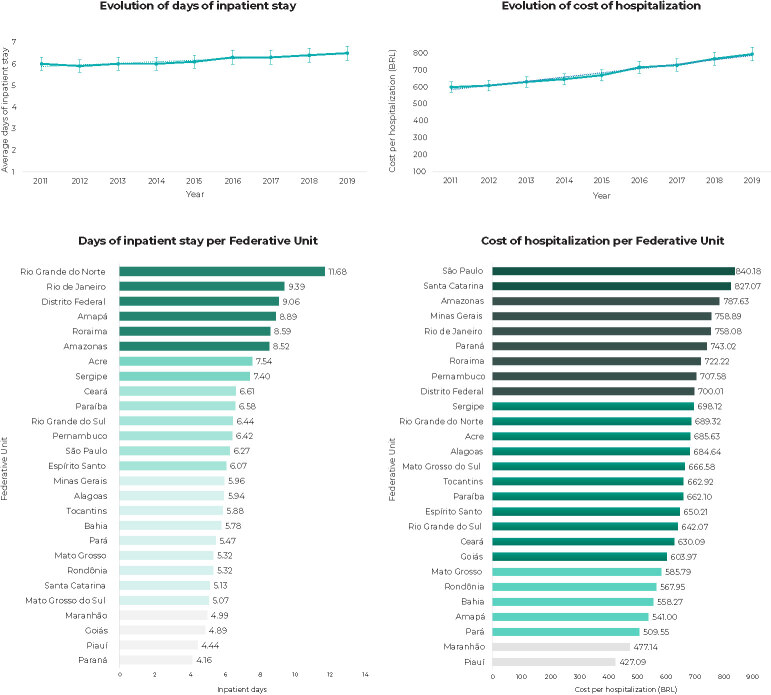
Overall trend panel, ranking per Federative Unit and territorial thermometer of days of inpatient stay and costs of hospitalization for cases having diabetes *mellitus* as main diagnosis in the Brazilian National Health System, Brazil, 2011-2019

In the study period, the national average for days of hospitalization due to DM was 6.17 days, being lower in the Southern (5.32) and Midwest (5.70) regions (Figure 2). The longest length of stay was found in the states of Rio Grande do Norte (11.68), Rio de Janeiro (9.39), Distrito Federal (9.06), Amapá (8.89), Roraima (8.59) and Amazonas (8.52), while the lowest length of stay was recorded in the state of Paraná (4.16). In Brazil as a whole, average length of stay had a rising trend, going from 6.00 days in 2011 to 6.50 days in 2019 (Table 2). 

**Table 2:** Average and trend of length of stay and hospitalization rate (HR) for cases having diabetes *mellitus* as main diagnosis in the Brazilian National Health System, by sex, race/skin color, age group and age range per 10,000 inhabitants in Brazil, 2011-2019

Brazil	Time 2011	Time 2019	Average 2011-2019 (95%CI)	APC (95%CI)	p-value	Trend
Length of stay	6.00	6.50	6.17 (6.00;6.3)	3.90 (1.30;0.27)	0.002	Rising
**HR according to Characteristics**	**HR** **2011**	**HR** **2019**	**Average 2011-2019 (95%CI)**	**APC** **(95%CI)**	**p-value**	**Trend**
**HR per sex**						
Female HR	8.36	6.31	7.12 (6.52;7.72)	-0.46 (-0.56;-0.34)	< 0.001	Falling
Male HR	6.69	6.65	6.43 (6.29;6.58)	-0.48 (-0.69;-0.13)	0.020	Falling
**HR per race/skin color**						
White	4.90	3.83	4.09 (3.79;4.38)	-0.57 (-0.76;-0.35)	< 0.001	Falling
Black	4.03	4.03	3.67 (3.49;3.85)	-0.57 (-0.75;-0.28)	0.010	Falling
Mixed race	205.05	230.16	211.21 (202.79;219.62)	536.03 (0.15; 25,7038.58)	0.040	Rising
Asian	0.08	0.57	0.26 (0.10;0.43)	0.02 (-0.21; 0.32)	0.830	Stationary
Indigenous	1.80	3.96	2.54 (2.05;3.02)	0.82 (-0.11;2.72)	0.090	Stationary
**HR by age range**						
**Children and Adolescents**	**1.23**	**1** **.55**	**1.35 (1.27;1.43)**	**0.10 (0.05;0.15)**	**< 0.001**	**Rising**
0-4 years	0.72	0.76	0.73 (0.71;0.75)	0.00 (0.00;0.02	0.090	Stationary
5-9 years	1.07	1.15	1.09 (1.06;1.12)	0.02 (0.00;0.05)	0.010	Rising
10-14 years	1.73	2.4	1.96 (1.82;1.17)	0.20 (0.15;0.26)	< 0.001	Rising
15-19 years	1.43	1.9	1.57 (1.45;1.7)	0.15 (-0.02;0.35)	0.090	Stationary
**Young adult**	**2.18**	**2.01**	**1.98 (1.89;2** **.07)**	**-0.29 (-0.38;-0.19)**	**< 0.001**	**Falling**
20-24 years	1.43	1.63	1.46 (1.40;1.53)	0.05 (0.00;0.12)	0.070	Stationary
25-29 years	1.68	1.68	1.58 (1.51;1.64)	-0.24 (-0.34;-0.13)	< 0.001	Falling
30-34 years	2.25	1.97	2.02 (1.89;2.15)	-0.29 (-0.46;-0.07)	0.020	Falling
35-39 years	3.33	2.76	2.86 (2.67;3.05)	-0.46 (-0.65;-0.19)	0.010	Falling
**Adult**	**11.01**	**8.34**	**9.21 (8.43;9.99)**	**-0** **.86 (-0.93;-0.72)**	**< 0.001**	**Falling**
40-44 years	4.91	3.9	4.26 (3.92;4.59)	-0.26 (-0.38;-0.13)	< 0.001	Falling
45-49 years	7.90	6.18	6.46 (5.70;7.23)	-0.13 (-0.54;0.66)	0.640	Stationary
50-54 years	12.55	9.7	10.47 (9.67;11.27)	-0.91 (-0.97;-0.66)	< 0.001	Falling
55-59 years	18.70	13.58	15.32 (13.84;16.81)	-0.78 (-0.90;-0.52)	< 0.001	Falling
**Young-old**	**37.68**	**25.2**	**30.31 (26.00;33.61)**	**-0.97 (-0.99;-0.92)**	**< 0.001**	**Falling**
60-64 years	26.08	18.16	21.24 (19.15;23.33)	-0.99 (-1.00;-0.91)	< 0.001	Falling
65-69 years	33.81	23.62	27.54 (24.08;30.28)	-1.00 (-1.00;-0.97)	< 0.001	Falling
70-74 years	41.55	27.18	33.75 (29.72;37.78)	-0.99 (-0.99;-0.97)	< 0.001	Falling
75-79 years	49.26	31.83	38.68 (34.26;43.11)	-0.99 (-1.00;-0.96)	< 0.001	Falling
**Oldest-old (80+)**	**52.23**	**30.97**	**40.53 (34.** **90;46.17)**	**-1.00 (-1.00;-1.00)**	**< 0.001**	**Falling**

HR: Hospitalization rate (per 10,000 inhabitants); 95%CI: 95% confidence interval; APC: Annual percentage change.

The amount spent on hospitalizations due to DM in the period totaled BRL 844,795,917 (the corrected amount being BRL 1,077,494,419 and US$ 420,692.23), while average annual expenditure was BRL 93,866,212 (the corrected amount being BRL 119,721,602 and US$ 46,743.58), while the average annual cost of hospitalization was BRL 681.52 (the corrected amount being BRL 867.63 and US$ 294.30), and was highest in the Southeast region. Figure 2 shows the average amount for the period by FU. The amount spent per hospitalization showed a rising trend over the period, increasing from BRL 598.83 in 2011 to BRL 793.01 in 2019 (p-value < 0.001).

## DISCUSSION

In the period 2011-2019, hospitalization rate due to DM showed a falling trend in most regions of Brazil, with the national average being 6.77/10,000 inhabitants, while it was higher in the Northeast (7.91) and Southern (7.61) regions, and lowest in the Southeast region (5.6). Hospitalization rates were higher for females and the elderly and showed a rising trend in the child and adolescent group (aged 5 to 14 years) and in the group of mixed race people. During the period, average length of stay was 6.17 days and total cost of hospitalizations was US$ 420,692,238.20, both of them having a rising trend. 

The disparities found between the Brazilian regions with regard to hospitalization rate due to DM have also been found in relation to other indicators, such as DM prevalence. A national survey carried out in 2019 found variation in the prevalence of self-reported DM between regions of the country, being lowest in the Northern region and highest in the Southeast, with a rising trend in the period 2013-2019.^7^ Regarding hospitalization rate, rates were higher in the Northern and Northeast regions, remaining above the national average, while being lower in the Southeast. The contrast between these data suggests divergences in access to healthcare: although the increased number of cases of the disease could increase future demand for hospitalizations, it is assumed that in regions with higher self-reported prevalence, early diagnosis and adequate treatment of the disease would allow control timely, avoiding complications and hospitalizations^7^ while in regions with lower self-reported prevalence there may be cases that are undiagnosed, untreated and that become severe, requiring hospitalization. 

Most of the regions and FUs that had high hospitalization rates due to DM are considered to have high social vulnerability. Income inequality is highest in the Northeast, according to the GINI index,^5^ and it had the highest hospitalization rate due to DM. Among the FUs, Maranhão and Piauí, which have Brazil’s lowest Human Development Index,^8^ had the highest hospitalization rate due to DM in the period, suggesting the influence of socioeconomic conditions on the occurrence of this event. A study carried out in the state of Pará, between 2008 and 2017, showed that regions where access to services was difficult and social indicators were low had the highest hospitalization rates due to DM.^8^ A study conducted in the state of Paraná showed a decrease in DM hospitalizations between 2005 and 2015, a period in which the Family Health Strategy (*Estratégia Saúde da Família* - ESF) was expanded.^9^

Despite the increase in DM prevalence and incidence in Brazil and worldwide, when analyzing the trend of hospitalization rate due to DM in the SUS, throughout the period studied, a falling trend was found in most regions and a stationary trend was found in the Northern region. When analyzing this result, it is important to consider the influence of the extent of SUS DM hospitalizations in relation to the total number of hospitalizations for this cause nationwide; the implementation of public policies, focusing on strengthening Primary Health Care (PHC); and medical demography in Brazil. 

According to the Oswaldo Cruz Foundation Observatory of Hospital Policy and Management (*Observatório de Política e Gestão Hospitalar* - OPGH), there was a drop in the SUS share of total hospitalizations in Brazil, from 74% in 2015 to 69% in 2019.^10^ Data from the 2019 National Health Survey (*Pesquisa Nacional de Saúde* - PNS) indicated that seven out of every ten people sought care in the public health network and that 28.5% of the Brazilian population (59.7 million people) had some form of health insurance, with greater coverage in the Southeast and Southern regions. This shows the dependence of the Brazilian population on public health services in general, since 71.5% of the population does not have access to supplementary healthcare via health insurance,^11^ especially in regions and states where the highest rates and/or rising trends of hospitalization rate due to DM were found.

In recent years (2006-2019), public policies have been reformulated and implemented with the aim of improving access to health services, such as the Pact for Health (*Pacto Pela Saúde*) established in 2006, the National Health Policy for the Elderly (*Política Nacional de Saúde da Pessoa Idosa* - PNSPI) with effect from 2006, the National Primary Care Policy (*Política Nacional de Atenção Básica* - PNAB), created in 2006 and reformulated in 2011, 2017 and 2019, and the EFS, in addition to the Action Plan do Address NCDs.^12^ This movement is important, as the chronic disease hospitalization rate is sensitive to the increase or reduction in PHC coverage. In the North, for example, despite the expansion of ESF coverage between 2013 and 2019, unmet health needs still persist among residents of that region compared to other Brazilian regions,^13^ which may have influenced the hospitalization rates found, as well as the hospitalization rate trend for that region.

Regarding medical demography, we noted that the Federative Units that showed an increase in hospitalization rate due to DM had some of the lowest medical coverage in the country, such as Piauí (1.6 doctor/1,000 inhabitants), Amazonas (1.3), Maranhão and Pará (1.1).^14^ On the other hand, in FUs in the Southern and Southeast regions, even with lower PHC coverage, there is a greater supply of health professionals, in addition to greater access to health services through private health insurance,^15^ allowing adequate prevention of health problems in these populations. 

The increasing trend in hospitalization due to DM as age increases, as found in this study, follows the global trend. There was a trend towards increased hospitalizations due to DM in the 5-14 year old population. Although in this study we did not assess specific types of DM, it is assumed that type 1 diabetes predominates in this age group, with higher incidence in those aged 10-14.^16^ The literature indicates growing rates of type 1 DM worldwide,^16,18^ frequently diagnosed at the time of admission to hospital.^17^

In this study, the number of hospitalizations due to DM was proportionally higher among the elderly group, especially in the 60-64 age range. This finding reflects the aging population and the increase in the diagnosis of people with DM2, which is based on factors related to lifestyle, resulting from greater urbanization, growing prevalence of obesity and sedentary lifestyle, as well as increased survival among people who suffer from diabetes.^19,20^

People of mixed race/skin color had the highest hospitalization rates and an increasing trend in hospitalizations due to DM. A study that analyzed the risk of developing diabetes during lifetime revealed that self-declared Black/mixed race Brazilians had a 7.5% higher risk of having diabetes in their lifetime when compared to those of White race/skin color, with this difference being greater among females. According to those estimates, a fifth of young Brazilians of White race/skin color and a quarter of those of Black/mixed race/skin color will develop diabetes throughout their lives. This information reinforces the importance of considering health disparities in relation to race/skin color when planning health promotion and disease prevention programs.^21^ It should be noted that, in this study, this data was missing in practically a third of the records, which may have affected the calculation of the measurements, although this proportion of losses for this variable is similar in primary research.^22-24^

Average length of stay (in days), as well as hospitalization costs due to DM, increased in Brazil during the period analyzed. It is known that DM length of stay tends to be longer^25^ and that the following factors are associated with longer stays: being of the male sex, age (60 or over), low level of education and previous diabetes decompensation, with a higher risk of readmission within 30 days.^26^ On the other hand, hospitalization offers an opportunity to address education for self-management of diabetes,^27^ given that educational actions about diabetes offered to decompensated individuals are associated with a 34% reduction in the likelihood of hospitalization for all causes within 30 days and a 20% reduction in the odds of readmission within 180 days.^28^ Likewise, studies have shown that educational approaches aimed at people with diabetes can contribute to reducing the cost of care, in addition to improving quality of life in the long term.^29,27,30^

This study has limitations common to ecological studies, such as use of secondary data, which may be incomplete or underestimated; it was not possible to assess hospitalizations according to the type of DM (type 1, type 2 and others), taking ICD codes E10-E14 grouped together. Furthermore, we considered hospitalizations of cases with diabetes as the main diagnosis, which may not have included hospitalizations due to complications resulting from DM, when recorded. The underlying cause of hospitalization is also subject to inadequate recording, which may underestimate the real impact of the disease on hospitalizations. In future studies, the ICD codes of the main complications of the disease can be added, including other conditions such as gestational DM that also cause hospitalizations, and/or applying more complex methodologies such as attributable risk, in order to cover all hospitalizations related to DM. It was also not possible to assess readmissions and transfers, and so duplication may have occurred. Furthermore, it is known that serious cases require referral services, which can generate more care in these locations, generally large urban centers. However, by presenting the data not only by FU but also by larger aggregates, namely Brazil’s five regions, we sought to minimize this distortion and highlight the inequalities that exist in the country. As to assessment of the race/skin color aspect, the large percentage of data without information compromises the characterization of this variable. It should be noted that our analyses only refer to SUS hospitalizations, and it would be important to complement them with information from the supplementary health system. In relation to expenditure, it is known that the SUS service price table has not been readjusted, since Constitutional Amendment 95/2016 froze public health service investments for 20 years, therefore the expenditure presented refers to approximate values, even though monetary correction was applied to the total amounts.

In short, SUS hospitalizations due to DM showed a falling trend in Brazil, with an increase in hospitalizations in the 5 to 14 year age range. The total financial impact exceeded 1 billion BRL (more than US$ 420 million), and this trend was rising. Even though hospitalization rates showed a falling trend, the increase in hospitalization time and expenditure suggests that hospitalizations occur in more serious and decompensated cases, and indicates a worrying scenario for the coming decades in Brazil, due to the epidemiological scenario of the disease. It is worth noting that hospitalization rates are impacted by socioeconomic issues, among other factors that hinder or facilitate access to health care. As such, the results of our study can provide support for the planning and organization of health care actions for people with diabetes *mellitus.*

## SUPPLEMENTARY MATERIAL

**Supplementary Table:** Evolution of diabetes hospitalization rates in Brazil, by Region/Federative Unit and sociodemographic characteristics, 2011-2019

Hospitalization rate per Region and Federative Unit/10,000 inhab.	2011	2012	2013	2014	2015	2016	2017	2018	2019
**Brazil**	**7.** **55**	**7.17**	**7.04**	**6.92**	**6.78**	**6.** **24**	**6.39**	**6.44**	**6.48**
**Northern Region**	**7.40**	**7.40**	**7.65**	**7.62**	**7.18**	**6.76**	**7.02**	**7.52**	**7.58**
Rondônia	13.24	12.31	13.45	13.11	11.20	10.24	11.03	11.74	10.06
Acre	6.53	5.86	6.19	5.67	5.18	5.05	4.81	5.82	5.14
Amazonas	4.85	5.75	5.33	4.42	5.84	5.68	6.34	7.47	6.99
Roraima	9.07	7.43	10.00	12.63	11.45	10.25	10.33	10.41	12.38
Pará	6.84	6.83	7.32	7.79	7.17	6.77	7.08	7.26	7.81
Amapá	6.33	6.40	6.19	4.91	3.76	3.37	3.27	3.57	3.29
Tocantins	10.72	10.44	9.61	9.47	7.59	7.10	5.98	6.28	6.89
**Northeast Region**	**8.89**	**8.15**	**8.11**	**8.24**	**8.38**	**7.31**	**7.53**	**7.55**	**7.64**
Maranhão	9.25	8.91	11.10	12.02	12.56	10.94	12.91	13.73	14.83
Piauí	12.20	12.67	12.62	12.66	13.28	11.94	10.71	10.57	10.27
Ceará	6.56	5.73	5.17	5.18	5.03	4.79	4.78	5.21	5.58
Rio Grande do Norte	8.88	7.35	6.68	6.82	7.63	8.45	7.86	8.30	6.68
Paraíba	9.05	7.58	7.13	7.40	8.10	7.23	7.57	7.49	6.74
Pernambuco	8.51	7.67	6.93	6.29	5.78	5.51	5.69	5.73	5.76
Alagoas	9.42	8.12	7.58	6.28	6.03	6.29	5.96	4.95	5.33
Sergipe	4.05	3.43	3.30	3.74	4.75	4.73	5.38	5.04	4.27
Bahia	10.19	9.58	9.67	10.25	10.34	7.64	7.72	7.37	7.61
**Southeast Region**	**6.08**	**5.88**	**5.76**	**5.58**	**5.51**	**5.21**	**5.41**	**5.45**	**5.51**
Minas Gerais	7.88	7.77	8.20	7.79	7.53	7.14	7.59	7.69	7.82
Espírito Santo	6.69	6.61	6.38	6.43	6.79	6.37	5.99	5.84	6.01
Rio de Janeiro	5.59	5.30	4.67	4.62	4.69	4.27	4.24	4.33	4.52
São Paulo	5.37	5.15	4.98	4.83	4.77	4.57	4.79	4.82	4.78
**Southern Region**	**8.73**	**8.** **53**	**8.10**	**7.84**	**7.45**	**7.13**	**7.** **04**	**6.98**	**6.81**
Paraná	8.71	8.71	8.46	7.54	6.86	6.48	6.91	6.92	6.92
Santa Catarina	7.01	6.72	6.36	6.03	6.67	6.92	6.42	6.43	6.22
Rio Grande do Sul	9.78	9.43	8.79	9.22	8.50	7.92	7.55	7.38	7.08
**Midwest Region**	**8.73**	**7.98**	**7.58**	**7.** **08**	**6.32**	**5.85**	**5.84**	**5.67**	**5.** **78**
Mato Grosso do Sul	7.36	7.25	7.12	6.63	6.45	6.33	6.99	7.70	7.40
Mato Grosso	8.90	8.43	7.70	7.78	7.42	6.60	6.09	5.69	5.77
Goiás	9.76	8.43	8.19	7.54	6.36	5.50	5.57	4.97	5.22
Distrito Federal	7.46	7.09	6.44	5.63	4.84	5.37	5.10	5.37	5.59
**Length of stay (days)**	**6.** **00**	**5.90**	**6.00**	**6.00**	**6.10**	**6.** **30**	**6.30**	**6.40**	**6.50**
**Total cost per Brazilian National Health System DM hospitalization (BRL)**	**598** **.83**	**607.91**	**629.40**	**645.02**	**668.54**	**715** **.43**	**728.04**	**765.64**	**793.01**
**Hospitalization rate per sex (10,000 inhab.)**	**2011**	**2012**	**2013**	**2014**	**2015**	**2016**	**2017**	**2018**	**2019**
Female	8.36	7.90	7.68	7.44	7.16	6.36	6.45	6.42	6.31
Male	6.69	6.41	6.37	6.38	6.38	6.12	6.34	6.46	6.65
**Hospitalization rate per race/skin color/10,000 inhab.**									
White	4.90	4.47	4.17	4.08	3.96	3.76	3.80	3.81	3.83
Black	4.03	3.67	3.55	3.48	3.51	3.36	3.66	3.75	4.03
Mixed race	205.05	199.66	205.64	216.95	216.19	196.48	208.76	221.98	230.16
Asian	0.08	0.06	0.07	0.06	0.19	0.36	0.44	0.53	0.57
Indigenous	1.80	2.14	2.46	2.58	2.58	1.98	2.45	2.87	3.96
**Hospitalization rate per age range/10,000 inhab.**									
**Children and Adolescents**	**1** **.23**	**1.24**	**1.28**	**1.31**	**1.34**	**1** **.31**	**1.41**	**1.46**	**1.55**
Up to 4 years	0.72	0.72	0.70	0.79	0.72	0.71	0.74	0.74	0.76
5-9 years	1.07	1.05	1.09	1.03	1.07	1.09	1.13	1.14	1.15
10-14 years	1.73	1.71	1.79	1.93	2.03	2.02	2.17	2.18	2.40
15-19 years	1.43	1.48	1.54	1.49	1.53	1.42	1.60	1.78	1.90
**Young adult**	**2.18**	**2.09**	**2.04**	**1.99**	**1.93**	**1.79**	**1.86**	**1.94**	**2.01**
20-24 years	1.43	1.38	1.42	1.46	1.45	1.38	1.49	1.54	1.63
25-29 years	1.68	1.61	1.60	1.58	1.54	1.42	1.51	1.56	1.68
30-34 years	2.25	2.23	2.18	2.00	1.98	1.80	1.86	1.91	1.97
35-39 years	3.33	3.13	2.95	2.91	2.74	2.57	2.59	2.76	2.76
**Adult**	**11.01**	**10.24**	**9.82**	**9.52**	**9.15**	**8.27**	**8.31**	**8.24**	**8.34**
40-44 years	4.91	4.87	4.49	4.42	4.11	3.80	3.92	3.88	3.90
45-49 years	7.90	7.42	7.26	7.16	6.79	6.20	6.01	6.25	6.18
50-54 years	12.55	11.47	10.91	10.59	10.29	9.43	9.78	9.49	9.70
55-59 years	18.70	17.19	16.61	15.91	15.40	13.64	13.54	13.35	13.58
**Young-old**	**37.68**	**34.59**	**33.15**	**31.67**	**30.36**	**27.07**	**26.74**	**26.30**	**25.20**
60-64 years	26.08	24.30	22.80	21.70	20.88	19.16	19.23	18.85	18.16
65-69 years	33.81	31.29	29.97	28.23	27.22	24.85	24.73	24.18	23.62
70-74 years	41.55	39.07	37.99	36.43	33.87	30.10	29.07	28.49	27.18
75-79 years	49.26	43.70	41.84	40.32	39.47	34.16	33.92	33.66	31.83
**Oldest-old (80+)**	**52.23**	**47.60**	**45.80**	**43.69**	**41.13**	**35.30**	**35.18**	**32.91**	**30.97**
