# ERRATUM: Time trend and costs of hospitalizations with diabetes mellitus as main diagnosis in the Brazilian National Health System, 2011 to 2019

**DOI:** 10.1590/S2237-96222024v33e2024056.en

**Published:** 2024-03-15

In the article *Time trend and costs of hospitalizations with diabetes mellitus as main diagnosis in the Brazilian National Health System, 2011 to 2019*, doi: 10.1590/S2237-96222023000400006.en, published on Epidemiology and Health Services, 32(4):e2023509, 2023, in the page 6, Figure 1:

Original text:

**Arrow indicating an increasing trend on the map in the state of Piauí (average rate 11.87).**

Corrected text:

**Arrow indicating an increasing trend on the map in the state of Maranhão (average rate 11.83).**

